# Observation and assessment of the nutritional quality of ‘out of school’ foods popular with secondary school pupils at lunchtime

**DOI:** 10.1186/s12889-017-4900-9

**Published:** 2017-11-17

**Authors:** Fiona Crawford, Dionne Mackison, John D. Mooney, Anne Ellaway

**Affiliations:** 1 Glasgow Centre for Population Health, Glasgow, UK; 2 Department for International Development, London, UK; 3 University of Sunderland, Sunderland, UK; 4 MRC/CSO Social & Public Health Sciences Unit, University of Glasgow, Glasgow, UK

**Keywords:** Food outlets, School pupils, Unhealthy diet, Schools

## Abstract

**Background:**

The contemporary Scottish diet is unhealthy and a risk factor for poor health outcomes including obesity. Over a third of Scottish children are at risk of being overweight or obese, and there have been calls to strengthen the evidence base on the role of the food retail environment around schools in influencing the consumption of unhealthy foods.

**Methods:**

We examined the food retail environment around five secondary schools in Glasgow city, Scotland. Trained fieldworkers observed the food purchasing behaviour of school pupils in local shops. Samples of the most popular foods were subsequently purchased by the research team and assessed for nutritional content, including energy, total and saturated fat, and salt. This was compared with the nutrient standards for school lunches established by the Scottish Government.

**Results:**

There was marked variation in the number of outlets identified within a 10 min walk from each school, ranging from five in the area with the lowest number of outlets to thirty in the area with the highest number of outlets. Outlets identified were heterogeneous and included fish and chip shops, kebab shops, convenience stores, newsagents, bakeries, mobile catering units, cafés, pizzerias, sandwich shops and supermarkets. Lunchtime offers and other marketing strategies targeting school pupils were observed at most outlets. Nutritional analysis of the 45 savoury food items purchased was conducted by laboratory staff. Of the foods analysed, 49% of the samples exceeded recommended calorie intake, 58% exceeded total fat recommendations and 64% exceeded saturated fat recommendations, 42% exceeded recommended salt levels. Over 80% of the 45 food items sampled did not comply with one of more of the nutrient standards for fat, saturated fat and salt. Meal deals and promotions of unhealthy foods aimed at pupils were widely available.

**Conclusions:**

The majority of pupils purchased unhealthy convenience food of poor nutritional value at lunchtime in local shops around their school. Further effort is required to implement regulatory levers such as taxation on unhealthy foods, restriction on the concentration of outlets selling unhealthy foods as well as the development of partnerships and additional measures within and beyond schools to promote healthy foods.

## Background

The contemporary Scottish diet is widely regarded as unhealthy and a risk factor for being overweight or obese as well as developing a number of chronic diseases including cancer, diabetes, and cardiovascular disease [1, 2]. Poor nutrition amongst Scottish children and young people remains a major public health concern [3]. Recent data indicate that nearly a third of children between the ages of two and 15 years of age are at risk of being overweight or obese while the proportions of adults who are overweight or obese has increased steadily over the last 20 years [4]. There is some evidence that weight status in childhood and adolescence tracks into adulthood with associated poorer health conditions in later life [5]. Whilst for Scotland as a whole, being at risk of obesity may be slowly declining; the rate of reduction is much more marked for children from affluent areas with a projected marked increased risk for children from the most deprived areas [6]. It has been suggested that healthy nutrition interventions should target young children and adolescents as these ages represent two critical time points for preventing or reversing potential adverse effects of overweight and poor eating habits later in life [7].

The school environment is regarded as a key setting in which to provide and promote healthy nutrition amongst children and young people. In Scotland, the school lunchtime environment and school meal standards have improved through policy and legislation. Nutritional standards are now in place in all state-funded educational establishments [8]. Concerted efforts have also been made to promote healthy nutrition amongst primary and secondary school pupils through evaluating approaches such as stay-on-site policies [9] and through the Scottish Curriculum for Excellence [10]. The introduction of secondary school lunchtime stay-on-site policies in Glasgow followed earlier research conducted in 2007 which concluded that different approaches were needed for primary and secondary school pupils and that measures to encourage secondary pupils to stay in school and eat a school lunch should be tested out [11].

Although school meal uptake has increased in Scottish primary schools during the last decade, secondary school meal uptake has decreased [12]; and in many urban areas across Scotland, secondary school pupils leave school premises at lunchtime to purchase food and drinks from external outlets in the local vicinity. The local vicinity around secondary schools in the UK has been researched and described by other commentators as the ‘school fringe’ or ‘school foodshed’ as it provides secondary school pupils with easy access to a diverse range of outlets that sell food and drinks, often of poor nutritional quality [13]. These outlets include take-away outlets, fish and chip shops, convenience stores, newsagents and supermarkets and are a characteristic feature of many Scottish urban high streets.

It has been argued that it is important to strengthen the evidence base in Europe regarding neighbourhood influences on food choices and nutrition [14]. Ellaway and colleagues examined whether different types of food outlets clustered around secondary schools across the city of Glasgow, Scotland [15]. They found an average of 35 food outlets per secondary school where energy dense foods could be bought at lunchtime. However, contrary to the evidence from other locations such as the USA [16] and urban areas in New Zealand [17], no clear pattern of clustering of food outlets near schools by socio-economic deprivation was found. Conclusions were that more attention needed to be given to the food retail environment around secondary schools in urban areas in order to build a better understanding of factors influencing pupils’ lunchtime food choices.

A small number of studies have examined the food environment around schools in relation to dietary intake with variable findings. One study in Germany [18] did not detect any relationship between food outlets and dietary intake whereas another study in Canada found that the presence of food retailers near schools was associated with pupils eating behaviour [19]. In the UK, a Scotland wide study found that two thirds of pupils purchased food and drink at out of school outlets during lunch time, with the prevalence higher among pupils from deprived areas [3].

However, few studies to date have assessed the nutritional content of foods commonly bought by pupils. This study therefore aimed to describe the commercial food environment near five secondary schools situated in Glasgow, Scotland, and to assess the nutritional quality of popular foods purchased by pupils using the Scottish nutrient standards for school lunches set by the Schools (Health Promotion and Nutrition) (Scotland) Act 2007 [20].

## Methods

### Study area selection

Five secondary schools were selected, representing a range of socio-spatial geographic segments of the city, varying size of school (based on pupil numbers), and socio-economic characteristics (represented by free school meal (FSM) entitlement. Parents/carers who are in receipt of an income-based benefit can apply for free school meals for their child.). A radius of 800 m (an approximate 10 min walk from the school gate) was delineated as the study area around each school for the purpose of examining the food environment. We used a cut off distance of 800 m representing a 10 min walk rather than 400 m representing a 5 min walk based on statistical methods initially employed by Austin et al. in their characterisation of school neighbourhood food environments in the US [21] and subsequently employed by Ellaway et al. in their analysis of food environments around socially disadvantaged schools in Glasgow [22]. A cut off distance of 800 m was assessed as appropriate in this study as Scottish secondary school pupils are entitled to a 40 min school lunch break and so have ample time to reach food outlets within an 800 m radius, purchase food and return to school if they walk briskly. Pupil numbers for each school ranged from 600 pupils in the smallest school to 1300 pupils in the largest. FSM entitlement varied from 12.2% to 42.2%. Average FSM entitlement across the city at the time of the research was 30%, double that for Scottish secondary schools.

### Mapping

Drawing on previous research [22] the density of food outlets around Glasgow secondary schools, the external commercial food environment in the five study areas was mapped using Geographic Information Systems (GIS) software and Glasgow City Council’s database which records the name, type and location of all commercial outlets selling food and drinks in Glasgow. Individual maps of each study area were produced showing the number and location of food outlets within a 10 min walk (c.800 m) from the school gates. An observational checklist was developed, piloted and refined, drawing on methodologies developed in similar research elsewhere [23, 24]. Ethical approval for the study was obtained from the University of Glasgow College of Social Sciences Ethics Committee for Non-Clinical Research Involving Human Subjects.

### Data collection

Preliminary observational scoping work in the vicinity of one of the study areas was conducted in May 2011 to establish the parameters for observational data collection. This included: identification of outlets observed as popular with pupils for the purchase of food and drinks; observation regarding length of queues at food outlets and types of food/drinks purchased; whether gender/age/ethnicity seemed to influence purchasing behaviour; and whether promotional offers (e.g. ‘two for one’, meals deals etc.) were offered by outlets. Following this scoping exercise a ‘purchase monitoring’ pro-forma was developed, piloted, refined and then utilised by members of the research team.

Between 10 am and 2 pm, on a designated Friday in September 2011, fifteen fieldworkers (two to four per study area, dependent on perceived volume of outlets) visited each of the five study areas to gather data on the characteristics of food outlets (e.g. sandwich shop, fish and chip takeaway, newsagents) and to observe which outlets appeared to be the most popular among pupils (assessed by queue length).

Fieldworkers distributed an information leaflet explaining the purpose of the research to shopkeepers/store managers in outlets prior to the data collection. A separate pupil information leaflet was also carried by researchers for issue to any pupil who became aware that s/he was being observed and asked for information.

Where possible, in addition to the use of the data collection checklist, fieldworkers engaged shopkeepers and outlet staff in conversation regarding items sold and what was perceived as popular with pupils.

Through observation in each of the five study areas during the 40 min lunch-time break, fieldworkers identified and listed popular savoury food items frequently purchased by secondary pupils. Following the fieldwork, each of the 5 fieldwork leads identified approximately 10 of the most commonly observed items that were purchased on the day of observation. As a result of this, 45 items were identified for purchasing for nutritional analysis. It was felt to be important to strike a balance between identifying the most popular items and reflecting the diversity of items seen purchased. Some study areas offered more choice in terms of outlets and therefore greater diversity of purchase options. In addition, a limited number of similar items (such as chips and curry sauce) were purchased from different outlets in order to compare their nutritional content. Chips and curry sauce (comprising chipped potatoes, similar to French fries, served with a curry flavoured savoury sauce) is a cheap, culturally acceptable and very popular lunchtime purchase widely available from many food outlets in Glasgow.

In collaboration with Glasgow City Council environmental health colleagues, sampling officers subsequently purchased the 45 pre-agreed items (one week later), recording the outlet from which the item was purchased, study area and cost of each item. Nutritional analysis of these items was conducted by Glasgow Scientific Services to compare the quality of key nutrients (energy, fat, saturated fat and salt) with Scottish nutrient standards for school lunches.

The nutrient standards for school lunches were established to ensure that a school lunch provides approximately one third of a pupil’s daily nutritional needs. When introduced, guidance for caterers on the implementation of the standards was provided. This guidance included advice on portion sizes to ensure that meals fulfilled appetites (and did not leave children and young people hungry or vulnerable to snack on confectionary or other high fat and sugar products). The nutrient standards for school lunches also set out the amount of energy, a minimum level for key nutrients and a maximum level for total fat, saturated fat, non-milk extrinsic sugars and sodium that must be provided in an average day’s school lunch (averaged over one week). For the purposes of this study, samples collected from external sources were compared with the Scottish nutrient standards for school lunches for energy (2776 kJ (664 kcals)), total fat (no more than 25.8 g) and saturated fat (no more than 8.1 g). Due to analytical techniques, the nutrient standard for Sodium (no more than 824 mg) was converted to salt (no more than 2 mg) and used as a comparator. Because the samples collected were primarily savoury (main items) data for non-milk extrinsic sugars are not provided.

## Results

### Characteristics of outlets

There was marked variation in the number of outlets identified within a 10 min walk from each school, ranging from five in the area with the lowest number of outlets to thirty in the area with the highest number of outlets. Outlets identified were heterogeneous and included fish and chip shops, kebab shops, convenience stores, newsagents, bakeries, mobile catering units, cafés, pizzerias, sandwich shops and supermarkets. Lunchtime offers, meal deals, price promotions and other marketing strategies targeted at school pupils were observed at many outlets.

### Pupil purchasing behaviour

Observers noted a brisk exodus from the school grounds by pupils when the lunchtime bell rang. Long queues of pupils quickly formed at popular outlets although there was a very rapid turnover in relation to items purchased. The most popular purchases contained chips/fries often with bread rolls, curry sauce, gravy, cheese, or fish etc. Other popular food items were sausage rolls, pizzas, individual servings of instant noodles, beef burgers/cheese burgers, rolls and sausage and doner kebabs. The price of purchases varied: the cheapest savoury item was a sausage roll costing 64 pence and the most expensive purchase was chips and a pizza slice costing £2.50. Many pupils were observed to augment their main purchase with additional items such as carbonated drinks, chocolate, crisps, and sweets. Some pupils took advantage of meal deals, although observations also suggested that school children were prudent shoppers who selectively purchased supplementary items (sweets and drinks in particular) from alternative outlets. Items purchased off site by pupils were assessed as lunch replacement rather than enhancement as pupils were observed leaving their schools immediately after the lunch time school bell rang and walking briskly to the shops, returning to schools shortly before the end of the lunch time break.

### Nutritional analysis

Laboratory staff from Glasgow Scientific Services conducted the nutritional analysis of the 45 savoury food items purchased. Some more popular items (such as chips and curry sauce) were obtained from different outlets to assess if their nutritional content differed.

Items were analysed for energy, fat, saturated fat and salt content and compared with the Scottish nutrient standards for school lunches in relation to each parameter. As foods sampled were intended for individual consumption (and given that there was marked variation in portion sizes across all purchases) nutrient analysis was presented per portion. Where analysis refers to per portion this refers to per portion as sold by the retailer. In addition, the calorific composition of foods was presented both per portion and per 100 g (per 100 g value was considered of interest to enable comparability of energy density across items where portion size varied greatly).

**Fig. 1:**
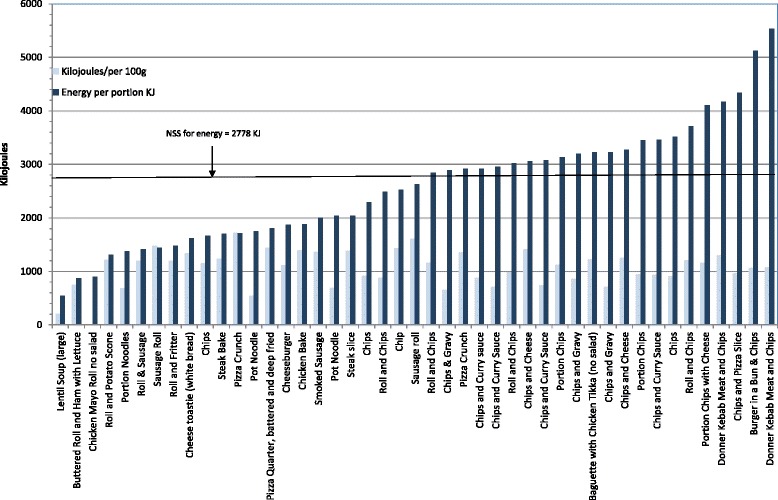
Energy content of savoury food items per portion and per 100 g

**Fig. 2:**
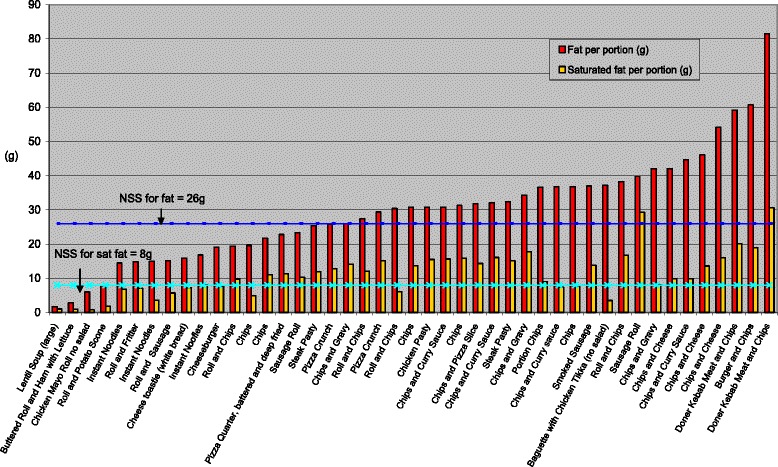
Total fat and saturated fat content per portion

**Fig. 3:**
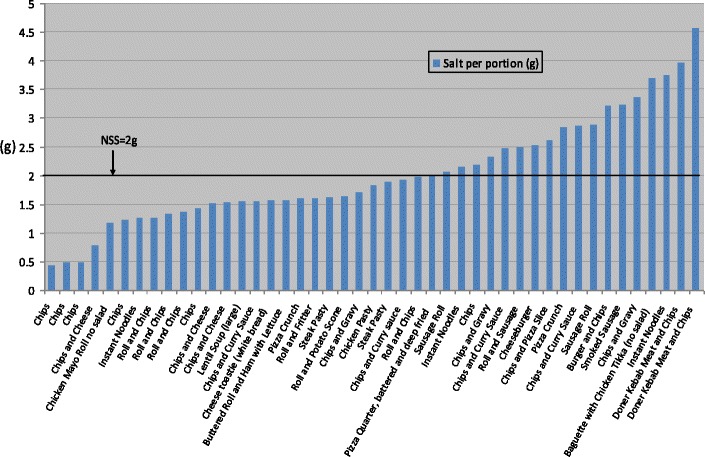
Salt content of savoury food items per portion (Grams)

Figure 1 displays the energy content of samples per portion (and per 100 g) and found that this ranged from 131 KCals to 1323KCals per portion. As can be seen in Figs. 2 and 3, fat and saturated fat content was similarly variable, ranging from 2 g to 80 g and 1 g to30g respectively while salt content varied from 0.4 g to 4.5 g. Twenty two of the 45 food items exceeded the recommended energy level for school lunch (664 KCalories); 26 exceeded recommended fat levels (26 g) and 29 exceeded saturated fat levels (8 g); 19 food items exceeded recommended salt levels (2 g). The majority (37) of the 45 food items sampled did not comply with one or more of the nutrient standards for fat, saturated fat and salt. Notably, 23 of the 45 food items did not meet the minimum energy levels of 664 KCalories.

As noted earlier, a large number of pupils were observed augmenting savoury food items with sweetened carbonated drinks, crisps, and confectionery thereby increasing the likelihood that their lunchtime energy, fat and salt consumption was greater than that revealed by the nutritional analysis. With the exception of a portion of lentil soup and a bread roll that contained lettuce, there was no inclusion of fruit, vegetables or salad in any of the samples analysed.

## Discussion

This was one of the first UK studies to gather observational data on school pupils’ external lunchtime purchases and provides insights into food commonly available and purchased beyond the school gate in an urban setting. None of the food outlets we observed in our study provided nutrition information on-site on the nutritional composition of their savoury food items. The nutritional analysis of popular savoury food items highlighted a stark contrast between the nutritional quality of the food available within school and that commonly sold by external commercial outlets near schools. The majority of those pupils observed eating out of school at lunch-time bought unhealthy, convenience food of very poor nutritional quality. A number of outlets selling food in the study areas were offering meal deals and promotions to pupils that contrasted sharply with the type of food available in school canteens. None of the meal deals or promotions offered fruit or vegetable/ healthy components. The concerns of staff and parents over the availability of food outlets around schools have been noted in previous evaluations of school lunchtime stay-on-site policies and programmes [25]. Aside from concerns over the potential consumption of unhealthy food and drink from these outlets, other issues included road safety, bullying, truancy, smoking, stranger danger etc.

Concern over the unhealthy diet and rising prevalence of obesity in children and adults have driven policy and the Scottish Government report ‘Preventing Overweight and Obesity in Scotland: A route map towards healthy weight’ set out plans and actions to prevent obesity at a population level [26]. A number of measures were recommended in the school setting such as supporting schools to encourage pupils to remain on site and exploring ways to restrict access by pupils to food outlets around schools. The Scottish Government’s Better Eating Better Learning policy launched in 2014 provides practical advice and examples of good practice to support schools, local authorities, caterers, procurement departments,parents, children and young people to work in partnership to make further improvements in school food and food education [27]. In addition, ‘Beyond the School Gate Guidance’ was also published by the Scottish Government in 2014 to support local authorities, schools, retailers, caterers and other partners in their efforts to influence the food environment around schools and support children and young people to make healthier choices [28].

Regulatory levers to discourage the consumption of unhealthy foods and drinks include health-related food taxes and these, alongside subsidies on healthy foods, have the potential to improve health [29]. Sales tax on sugared drinks, sweets and snacks has been introduced in the US, Australia and in several European countries. In the UK repeated calls to consider the introduction of taxes on unhealthy food and drinks to help tackle growing obesity levels have led to the announcement of the introduction of a sugar tax by the UK Government [30]. This, however, is only one potential lever that may impact on dietary behaviour. Taxation and other fiscal measures need to be part of a wider suite of public health initiatives that address other important determinants of food purchasing and consumption including accessibility, availability, affordability, acceptability and attitudes. Caraher et al. highlight the importance of recognising that if secondary pupils are treated solely as consumers and exposed to untrammelled fast-food marketing in locations near schools then they are very likely to purchase and consume unhealthy food and drinks [31]. Public health action needs to take place across educational, commercial, and local authority boundaries as well as through engagement and awareness raising work with parents and pupils. Tyrell et al.’s findings support these recommendations highlighting the fact that ‘takeaway and fast food’ environments are popular with young people and that current policies/initiatives to promote healthier food items will only ever have limited success if they continue to rely on voluntary agreements with the commercial food sector [32]. Small independent vendors also face barriers to selling healthy food choices to customers. A qualitative study exploring the views of independent fast food vendors near secondary schools in disadvantaged Scottish neighbourhoods found that vendors faced clear barriers to offering healthy food choices to customers. The study recommended that ‘Interventions and policies that target the food environment around schools should take the neighbourhood context into consideration’ [33].

The use of licensing and planning powers to limit the number and concentration of commercial outlets selling unhealthy food in local neighbourhoods and near schools is underway in a number of local authorities [34]. These powers could also be used in Scotland, building on current initiatives to avoid overprovision of alcohol outlets in particular areas and through public health input into local development plans. Moreover, the remit of local authority environmental health departments could be extended to include nutritional assessment/regulation.

As well as regulatory levers, collaborations with the commercial/business sector have been proposed. Policies such as Scotland’s National Food and Drink Policy [35], ‘Becoming a Good Food Nation’ [36] and ‘Supporting Healthier Choices’ [37] provide the policy foundations for industry collaborations to improve health. However, the extent to which voluntary collaborations and measures can have a positive impact on the Scottish diet remains to be seen.

### Limitations

This study had a number of limitations. Firstly, data were collected six years ago during one school-lunchtime in September. Therefore findings may not be representative of pupil behaviour during school lunchtimes on other school days in the week. Furthermore, eating patterns, particularly amongst pupils from lower socio-economic backgrounds may have changed over recent years in response to ongoing austerity and rising levels of child poverty in Scotland [38]. Seasonality and weather are also likely to affect numbers of pupils eating off-site and types of purchases. No data were captured from pupils themselves on the rationale for their outlet selection or their purchases (such as price, promotional offers in outlets, peer influence, etc). The research was unable to account for wastage of food purchased or for food sharing between pupils. It was also unable to determine the contribution made by lunchtime food to pupils’ overall dietary intake for the day. Finally, the research methods did not enable collection of any age/gender or socio-economic data of pupils eating off-site. Future research could address these limitations through more comprehensive observational and quantitative data collection and surveys that could also explore age/gender and socio-economic patterns more fully.

Also, school-based nutrient standards are based on the entire lunch-time meal and we are unlikely to have captured that with the items sampled. It is important to acknowledge that we have gained a snapshot of savoury foods and these may or may not be complemented with other items such as high energy, fat, and salt foodstuffs.

## Conclusions

The study has highlighted that there is ample opportunity for school pupils to purchase oversized energy dense products that are high in fat and salt during their school lunchtime break. The study findings also revealed scarce fruit and vegetable purchase and consumption.

Findings from this study provide clear evidence regarding the adverse impacts on pupils’ health and well-being of leaving school at lunchtime to purchase off-site food and highlight the need for greater availability of healthy, tasty, low cost food in external outlets. The maintenance and promotion of strict nutritional standards in relation to school based food and drinks are unlikely to bear fruit when such grossly unhealthy options are available and promoted just beyond the school gate. There is growing recognition of the importance of the physical and social environment in relation to children and young people’s food choices and behaviour. The Scottish Government’s ‘Better Eating Better Learning’ policy calls for a new context for school food that works more effectively in partnership with children, young people and their parents/carers to provide healthy, tasty lunchtime food in attractive physical and social school environments [27].This has especial relevance for low-income secondary school pupils who are entitled to free school meals but who may not be making use of their entitlement.

In 2012, the United Nations released a political declaration emphasising the need for the implementation of multi-sectoral, cost-effective, population-wide interventions in order to reduce the impact of unhealthy diets and other causes of non-communicable disease [39]. Lang and Rayner proposed that change would only be achieved by ‘big thinking, many changes’ [40]. They argued that coherence and optimism with firm political leadership across government, supply chains and civil society is essential. Creating healthier out of school food environments would be a good place to start.

## Abbreviations

FSM: Free school meals
GIS: Geographic Information Systems
Kcal: Kilocalories
Kj: Kilojoules
Mg: Milligram
